# Are Leadership Fairness, Psychological Distress, and Role Stressors Interrelated? A Two-Wave Prospective Study of Forward and Reverse Relationships

**DOI:** 10.3389/fpsyg.2018.00090

**Published:** 2018-02-06

**Authors:** Morten B. Nielsen, Jan O. Christensen, Live B. Finne, Stein Knardahl

**Affiliations:** National Institute of Occupational Health, Oslo, Norway

**Keywords:** role conflict/ambiguity, leadership, mental distress, longitudinal, work exposures

## Abstract

While previous research has mainly considered leadership as an antecedent to psychological distress and role stressors (i.e., role ambiguity and role conflict) among subordinates, a reverse relationship where these variables influence reports of leadership is also possible. To determine the directionality of the associations this two-wave prospective study assesses bidirectional relationships between fair leadership and role stressors and examines whether psychological distress mediates the reciprocal associations between fair leadership and the role stressors. Analyses were conducted in a sample of 6,790 Norwegian employees with a 2-year time-lag between measurement points. Fair leadership was associated with lower stability adjusted role ambiguity, but not role conflict, over time. Role conflict, but not role ambiguity, was related to subsequent reports of the immediate leader as less fair. Psychological distress did neither mediate the relationship between fair leadership and subsequent reports of role stressors, nor the association between role stressors and subsequent reports of fair leadership. The findings suggest that the fair leadership – role stressor association is not a one-directional process, but that exposure to role stressors also influence subordinates’ perceptions of leadership. An implication of the findings is that theoretical models of organizational leadership should include this reverse impact of role stressors. To reduce the effects of role stressors, organizations could set consistent, clear and attractive goals and provide employees with necessary information for conducting their work tasks in order to help workers understand and master their roles at the workplace.

## Introduction

In the context of the workplace, justice refers to employees’ perceptions of whether they have been treated fairly in their jobs and the ways in which those perceptions influence other organizational outcomes (Colquitt et al., 2005). Meta-analyses have shown that perceptions of justice are associated with both the psychosocial working conditions and the psychological health of employees (Cohen-Charash and Spector, 2001; Colquitt et al., 2013). As for the sources of justice, research has long recognized that the fairness of treatment received from superiors has an especially important influence on how the subordinates perceive their job (van Knippenberg et al., 2007; Long, 2016). Based on this line of reasoning, having a fair leader should contribute to favorable psychosocial working conditions and protect the psychological health of subordinates.

While this perspective of leadership is in line with the general assumption that the practices and behavior of leaders cause changes in subordinates, the idea of leadership as a mainly one-directional process should be questioned. Rather than treating fair leadership solely as an antecedent to employees’ psychological health and working conditions, we suggest that research must also consider the possibility of a reverse relationship where the subordinates’ perceptions of leadership fairness can be influenced by their working conditions and psychological health (Tang, 2014). In line with a follower-centered perspective on leadership (Shamir, 2007), subordinates are not merely passive recipients of leadership, but may also construct perceptions of leadership. It is probable that this perception process will be influenced both by characteristics of the work environment and psychological health. That is, while a fair leader may have an impact on how subordinates perceive work, other aspects of work and working conditions may also influence the subordinates’ perceptions of the leader as fair.

To examine such a reciprocity between leadership and psychosocial working conditions this two-wave prospective study examined bidirectional associations between fair leadership and role stressors in the form of role conflict and role ambiguity. Role ambiguity (antonym: role clarity) denotes uncertainty about the expectations, behaviors, and consequences associated with a particular role (Kahn et al., 1964; Rizzo et al., 1970), whereas role conflict refers to incongruence between differing expectations, either associated with one’s job role (“intra-role conflict”), different roles within a work context (“inter-role conflict”), or between job requirements and the employee’s opinions and ideals pertaining to how the job should be executed (Rizzo et al., 1970; Katz and Kahn, 1978).

Building on previous findings on justice and psychological health (Lang et al., 2011; Birkeland et al., 2016) and the notion of a rosy/gloomy perception mechanism (de Lange et al., 2005), we further propose that psychological distress is a mediating variable which explains how fair leadership and role stressors are bidirectionally interrelated. With regard to psychological health, psychological distress a non-specific term that refers to an overall negative emotional state experienced by the individual (Lovibond and Lovibond, 1995). Although psychological distress pertains to the dysfunctional aspects of psychological health, some degree of psychological distress is a common experience among workers (Strand et al., 2003). In the current study, we will therefore examine the normal variation in levels of psychological distress rather than as a clinical diagnosis.

Through examining reciprocal relationships as well as mediating mechanisms, the present study will extend previous research both methodologically and theoretically. The majority of existing studies on leadership have been based on cross-sectional designs (Shamir, 2011) which precludes testing the direction of associations. As for theory, previous research has largely ignored how the working conditions and psychological health of subordinates can influence their perceptions of leadership. Hence, by examining this kind of reverse association, the current study will contribute to our understanding of both the causes and consequences of fair leadership.

### The Impact of Fair Leadership on Role Stressors

While there are several dimensions of justice, research has traditionally focused on the two sub-domains distributive justice, which refers to perceptions of the fairness of decision outcomes and resource allocation; and procedural justice, which refers to perceptions of fairness regarding the processes leading to decision outcomes (Folger and Greenberg, 1985; van Knippenberg et al., 2007). In addition, the concept of interactional justice (also known as “relational” justice) has received increased attention in recent years (van Knippenberg et al., 2007). This form of justice refers to the dignity and respect with which one is treated, and to the extent in which one is timely, honestly, and accurately informed about personally relevant issues (Bies and Moag, 1986). Findings from meta-analyses highlights the role of the immediate supervisors as particularly important with regard to creating an experience of justice among employees (Colquitt et al., 2013). The results indicated that justice dimensions that were measured with reference to a supervisor as the source of justice were more strongly related to outcomes as compared to organization-focused justice perceptions, thereby providing strong support for the focus on supervisors as sources of justice (Colquitt et al., 2013). Justice seems to be important also in evaluations of leadership. For instance, an increased opportunity to express opinions has been found to heighten subordinates’ judgments of procedural justice and, thereby, subordinates’ evaluations of supervisors’ leadership capabilities, especially under conditions where the subordinates have low decision control (Tyler, 1986). Consequently, justice can be seen as an integral part of leadership (Zhang et al., 2014). As a form of leadership practice, leadership fairness may thereby refer to a supervisor distributing work fairly and treating workers fairly and equally (Dallner et al., 2000; Meierhans et al., 2008; Finne et al., 2014). While related to other forms of leadership such as transformational and ethical leadership, previous research has shown that (un-) fair leadership explain incremental variance in outcomes beyond levels of transformational leadership (Perko et al., 2016).

It has been suggested that perceived fairness of a role sender (e.g., leader) influences levels of the working conditions of employees, and especially role ambiguity and role conflict (Zohar, 1995). The “role episode” model by Kahn et al. (1964), “proposed that the experience of role ambiguity and role conflict would arise from the expectations and subsequent communications emanating from a role sender. Although expectations for a particular role incumbent may derive from other group members, it has been argued that the primary source of role-related expectations in a work setting is typically the immediate supervisor” (Beauchamp et al., 2005, p. 7). By setting clear and attractive goals and criteria for the achievement of these, leaders display a set of clear values that may help subordinates to understand the ends to which they are working (Nielsen et al., 2008; Bacha and Walker, 2013). For instance, by giving unambiguous orders and information and by helping with making priorities, the immediate leaders have a strong influence on subordinates’ role expectations. Consequently, with the leader as a role model, subordinates can observe how they can take responsibility for their own actions and development. Leaders may also influence followers’ role stressors by ensuring that they have the information required to conduct work tasks and by providing knowledge and support that enables them to develop skills required to conduct tasks (Nielsen et al., 2008). With regard to leadership fairness, this suggests that a leader who promotes fair treatment could reduce role ambiguity and role conflict. That is, role-modeling fairness in procedures and distributions of resources should contribute to reducing uncertainty regarding expectations and behaviors for a given role among subordinates and should also help increasing role clarity and reduce role conflict by ensuring that followers have the information required to conduct their work tasks.

While there are some previous studies confirming a relationship between different forms of leadership and variation in role conflict and role ambiguity (Shoemaker, 1999; Beauchamp et al., 2005; Skogstad et al., 2014), there are to our knowledge no previous studies that have investigated the specific impact of leadership fairness. However, research on organizational justice provides compelling evidence that fair treatment is associated with more desirable attitudes and behavior in response (van Knippenberg et al., 2007) and following the theoretical arguments for the relation presented above, we find it plausible to suggest that employees who experience fair leadership should report reduced role conflict and role ambiguity. The following hypotheses will therefore be tested:

H1a: Fair leadership is negatively related with subsequent role ambiguity.
H1b: Fair leadership is negatively related with subsequent role conflict.

### The Impact of Roles Stressors on Fair Leadership

Although most previous research has considered leadership as a causal factor with both work-related outcomes and employee health (Shamir, 2011; Nielsen et al., 2016), some has also claimed that the leader-subordinate interaction is a reciprocal process where subordinates also influence the behavior and practices of the leader (Schyns and Bligh, 2007; Shamir, 2011). Highlighting such a dual relationship, Burns (1978) described transformational leadership as a dynamic, two-way process in which both leaders and followers are being transformed by each other. In a similar manner, Shamir (2007) argued for a follower-centered perspective on leadership by claiming that followers are not merely passive recipients of leadership, but that subordinates may also construct perceptions of leadership. This impact of follower characteristics on ratings of leadership can be explained with the rosy/gloomy perception mechanism (de Lange et al., 2005; Tang, 2014). That is, depending on whether the employees experience the working conditions as positive or negative may determine whether they perceive their leader in a favorable or unfavorable light. Consequently, an employee who experience high levels of role conflict and/or ambiguity may attribute the causes of this exposure as his/her leader being unfair with regard to how the employee is treated (“My job role has not been thoroughly clarified by my superior”/”My leader has given me conflicting work tasks”).

While there are no previous studies that have empirically examined the potential impact of role stressors on fair leadership over time, the abovementioned arguments for a relationship between role stressors and subsequent ratings of the immediate leader as less fair lead to the following hypotheses:

H2a: Role ambiguity is negatively related with subsequent ratings of fair leadership.
H2b: Role conflict is negatively related with subsequent ratings of fair leadership.

### The Mediating Role of Psychological Distress

Above, we argued for rosy/gloomy perceptions as a mechanism that can explain how fair leadership and role stressors are interrelated. In the following, we will elaborate on how this mechanism, in conjunction with Lazarus and Folkman’s (1984) transactional model of stress, can explain how psychological distress function as an intervening factor in the two-way associations between fair leadership and role stressors. The rosy/gloomy perception mechanism suggests that levels of psychological distress can influence how workers perceive and attribute their work environment (de Lange et al., 2005; Tang, 2014). Specifically, gloomy perceptions means that distressed employees are likely to evaluate their environment more negatively and thus report less favorable work conditions. That is, these distressed workers have a gloomier perception of the external environment compared to non-distressed colleagues (de Lange et al., 2005). In an opposite manner, rosy perceptions suggest that healthier workers color their perceptions of work conditions in a rosier and more positive light, for instance because non-distressed workers can have more energy to work faster, and this energy can lead them to re-interpret their job demands as less demanding across time (de Lange et al., 2005). Supporting this mechanism, research in positive psychology has shown that psychological health in the form of optimism is a fundamental contributor to employee perceptions of work characteristics (Luthans et al., 2007). Hence, having an optimistic view on life may also lead to more rosy perceptions.

Based on Lazarus and Folkman’s (1984) transactional model of stress and coping, it can be argued that the impact of psychological distress on perceptions of working conditions may explain both how fair leadership may influence role stressors and how role stressors in reverse can explain reports of fair leadership. Work exposures such as unfair leadership, role conflict, and role ambiguity are stressors that can have detrimental effects on and the psychological health of employees. Whether an event is regarded as a stressor is, according to Lazarus and Folkman, determined by two consecutive appraisal processes (i.e., primary and secondary appraisal). In the primary appraisal process, the encountered situation or event is cognitively evaluated for its potential for harm or loss. If individuals perceive the situation as threatening, a secondary appraisal process is initiated, centering on whether one has available options or enough resources to meet the situational demands to prevent threat of harm or loss. If individuals perceive that the challenge of the situation is taxing or exceeding the available options and resources, the model proposes that individuals experience strain (Lazarus and Folkman, 1984). Strain over an extended time-period will manifest itself through psychological distress (e.g., anxiety, depression, exhaustion). As described above, psychological distress is a key factor with regard to gloomy/rosy perceptions.

**FIGURE 1:**
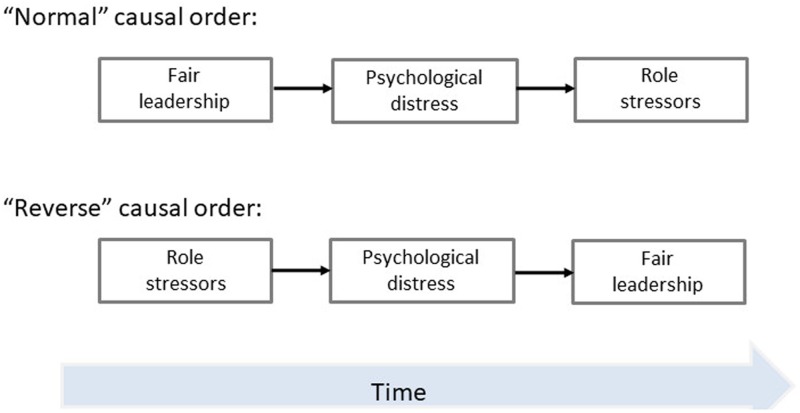
Two different pathways for the associations between leadership fairness, psychological distress, and role stressors.

The combination of the Lazarus & Folkman model and the gloomy/rosy perception model suggests two different pathways that can describe how leadership fairness relates to role stressors (**Figure 1**). In the first pathway, exposure to (un-)fair leadership represents a stressor that influence levels of psychological distress. Perceptions caused by levels of distress will thereby determine how employees experience the working conditions. That is, the experience of an unfair leader is expected to contribute to higher levels of distress that subsequently generate a more negative expression of the work environment, here reflected through role conflict and role ambiguity. In contrast, fair leadership should reduce or at least maintain low levels of distress and may thereby induce a more positive impression of the work environment. The second pathway takes a reverse outlook on the variables by considering role conflict and role ambiguity as stressors that influence levels of psychological distress. In resemblance with the first pathway, distress is the intervening variable and levels of distress is assumed to influence whether the employee perceive the leader as fair. Specifically, in line with the Lazarus and Folkman model, exposure to role conflict and role ambiguity is expected to increase levels of distress and this distress will make the employees perceive the leader as more unfair over time.

While there is a lack of previous longitudinal studies examining psychological distress as a mediator between leadership fairness and distal job related factors, there are some findings on related concepts indicating the likelihood of indirect associations between the variables (Spell and Arnold, 2007; Tang, 2014). In support of the second pathway, a systematic review of the reciprocal interplay between psychosocial job stressors and worker well-being, moderately strong evidence was established for a reverse effect of well-being on job demands (Tang, 2014). In primary studies, role conflict and role ambiguity has been longitudinally related to increased distress (Finne et al., 2014) and Birkeland et al. (2016) found that psychological distress was associated with perceiving the immediate leader as less fair over a 12 month time-period. Time-lagged associations between distress and later justice perception was also established in a three-sample study in a military context (Lang et al., 2011).

The potential impact of leadership fairness on working conditions and well-being has been examined in several cross-sectional studies (Sparr and Sonnentag, 2008; Tremblay, 2010). As for the potential impact of fair leadership in role stressors through distress, a cross-sectional study established that anger and fear mediated the relationship between perceived interactional justice and counterproductive work behaviors (Le Roy et al., 2012). In another cross-sectional study of indirect associations, the findings showed that ineffective supervision (abusive supervision and authoritarian leadership style) and employees’ distal negative outcomes (e.g., exhaustion, physical symptoms, job dissatisfaction, intention to quit, and poor job performance) were mediated by anxiety and depression (Pyc et al., 2017). In the latter study, it was concluded that there is a need for prospective studies which can examine the direct and indirect effects over time in order to better understand the causal order of the variables. In response to this call, and based on the above reasoning for potential indirect effects of distress in the associations between leadership fairness and role stress, we will test the following hypotheses:

“Normal” causal order (first pathway):

H3a: The negative relationship between fair leadership and subsequent role ambiguity is mediated by psychological distress.
H3b: The negative relationship between fair leadership and subsequent role conflict is mediated by psychological distress.
“Reverse” causal order (second pathway):
H4a: The negative relationship between role ambiguity and subsequent fair leadership is mediated by psychological distress.
H4b: The negative relationship between role conflict and subsequent fair leadership is mediated by psychological distress.

## Materials and Methods

### Procedure and Participants

This study is based on data from The New Workplace project which is a survey of Norwegian employees working in a full time or part time position (Christensen and Knardahl, 2010; Finne et al., 2014). In accordance with the requirements for health research in Norway, this project was approved by the Regional Committees for Medical and Health Research Ethics (REC) in Norway (REC South East), has permission from the Data Inspectorate of Norway and was conducted in accordance with the World Medical Association Declaration of Helsinki. Subjects were recruited from organizations based in Norway that were contacted and offered participation. Hence, at the organizational level, a convenience sampling technique was applied. The survey design was full-panel prospective with all variables measured at two time points. Average time-lag between baseline (T1) and follow-up (T2) was 24 months (range: 17–36 months). Meta-analytic findings have shown that a time-lag of 2–3 years is optimal with regard to detecting temporal occupational stressor-strain associations (Ford et al., 2014). According to Ford et al. (2014), a time-lag of 2–3 years is generally consistent with conservation of resources and allostatic load theories which suggests that cumulative exposure to chronic work stressors increases reactions to those stressors over time.

A variety of job types and organizations, including municipalities, insurance companies, health institutions, and public organizations, were represented in the survey. Participants could respond to the survey through an online electronic form or by filling out a pen-and-paper questionnaire. After excluding workers that were on absence, all employees were mailed a letter with information about the survey. In addition to information about the survey, informed consent, and ethical considerations, the enclosed letter provided the respondents with a personalized code for logging into the online questionnaire and the paper version of the questionnaire with a pre-stamped return envelope. It was emphasized in the letter that all responses would be treated confidentially, in strict accordance with the general guidelines and specific license from the Norwegian Data Inspectorate.

By August 2015, 31,942 employees from 97 organizations had been invited to participate at the baseline assessment. Altogether 16,143 responded (response rate: 51%), of which 91% responded to the survey using the electronic survey form. Altogether 14,586 of the baseline sample have been invited to the follow-up survey, with a total of 8,992 (62%) providing responses. In the current study, participants with more than 10% missing data on the items in the measurement inventories were excluded from the sample (Myers, 2011). In cases with less than 10% missing data, imputation was performed with the Hot Deck imputation procedure. With this method each missing value is replaced with an observed response from a participant with similar characteristic on pre-determined anchor variables (Myers, 2011). Age, gender, and leadership position were used as anchor variables in the imputation.

The final sample comprised 6,790 respondents. Mean age was 44.46 (*SD* = 10.1) years with a range from 19 to 69. The sample consisted of more women (55%) than men (45%). Four percent had between 1 and 9 years of education, 36% had between 10 and 12 years, 44% had between 13 and 16 years, and 16% had 16 years or more. Ninety-five percent had a regular full time employment, and 78% had a day work schedule. Altogether 23% had a leadership position that included personnel responsibility for subordinates.

Attrition analyses were conducted to examine whether the final study cohort differed from dropouts with regard to demographic characteristics, role stressors, distress, and fair leadership. Using data from the T1 assessment, the attrition analyses showed that the gender distribution was equal among sample and dropouts (X2 = 2.48; 1; *p* > 0.05). The study respondents (*M* = 44.39; *SD* = 10.09) were significantly (*t* = -9.2; *df* = 14,751, *p* < 001; Cohen’s *d* = 0.15) older than drop-outs (*M* = 42.91; *SD* = 11.82). With the exception of the finding that the study sample reported marginally lower levels of psychological distress (*t* = 4.77; *df* = 14,751, *p* < 001; Cohen’s *d* = 0.07), no significant differences were established between cohort and dropouts on the study variables at T1. The Cohen’s *d* values show that the actual differences between the respondents and drop-outs were small (Cohen, 1988). Hence, the T2 respondents seems to be representative of the overall sample.

### Instruments

#### Perceptions of Fair Leadership

Were measured by a previously validated scale from the General Nordic Questionnaire for Psychological and Social Factors at Work (QPS_Nordic_; Dallner et al., 2000; Wannstrom et al., 2009). The three items in the scale ask respondents to rate their immediate superior with regard to impartiality and equality in decision-making processes (i.e., “Does your immediate superior distribute the work fairly and impartially?,” “Does your immediate superior treat the workers fairly and equally?”). Answers were provided on a five point Likert scale: “1 = very seldom or never,” “2 = somewhat seldom,” “3 = sometimes,” “4 = somewhat often,” and “5 = very often or always.” The scale had acceptable internal consistency as measured with Cronbach’s alpha at both time-points (T1 = 0.80; T2 = 0.82).

Scales from the QPS_Nordic_ were used to measure *role ambiguity* (3 items, Cronbach’s alpha = 0.80/0.82) and *role conflict* (3 items, Cronbach’s alpha = 0.70/0.70). Example items for role ambiguity are “Do you know what your responsibilities are?” and “Do you know exactly what is expected of you at work?” Example items for role conflict are “Do you receive incompatible requests from two or more people?” and “Do you have to do things that you feel should be done differently?” Respondents provided their responses on a five point Likert scale ranging from “very seldom or never” to “very often or always.” The items for role ambiguity were originally phrased to reflect role *clarity*, and were therefore reversed in the current study.

#### Psychological Distress

During the last week was measured by the 10-item version of the Hopkins Symptom Checklist (HSCL-10). The HSCL is a psychometrically sound, and commonly used self-administered instrument designed to measure psychological distress in population surveys (Derogatis et al., 1974). The HSCL-10 is a reliable and valid representation of the full 25 item version (Strand et al., 2003). Responses were given on a four-point scale, ranging from “1 = not at all” to “4 = extremely.” Example items are “Suddenly scared for no reason” and “Feeling of worthlessness.” Cronbach’s alpha for this scale were 0.85 at T1 and 0.86 at T2.

#### Control Variables

The potential confounding influence of gender, age, and skill level was adjusted for in all analyses. Skill levels were determined based on classification of occupations according to a Norwegian adaptation of the International Standard Classification of Occupations (ISCO-88), developed by Statistics Norway. This classification reflects educational levels or equivalent levels of working experience required for different occupations according to the International Standard for Classification of Education. The five levels are: 1 = Occupations requiring the equivalent of a first or postgraduate university degree, or college exams based on a similar length of study (>16 years); 2 = Occupations normally requiring 1–3 years of university- or college-level education, but not equivalent to a first university (13–15 years); 3 = Occupations requiring 1–3 years of secondary education (10–12 years); 4 = Occupations requiring no more than 9 years of primary education; 5 = Occupations in which the level of required education vary substantially. Owing to the unspecified categories, the skill-level variable was treated as nominal by constructing dummy variables.

### Statistical Analyses

Relationships between role stressors, leadership and psychological distress were analyzed with Structural Equation modeling (SEM). Analyses were conducted in four steps. As a first step, the measurement models and the dimensionality of the latent variables at each time point were examined. As a second step, we investigated the measurement invariance across time for the latent variables. In the third step, structural models designed to address the directional associations between role stressors and fair leadership were specified and tested. Through specifying and testing a full cross-lagged autoregressive model, it was possible to contrast the causal directions between the variables. In the fourth step, psychological distress was investigated as a potential mediator of the relationship between role stressors and fair leadership by following the recommendations for a semi-longitudinal mediation model by Cole and Maxwell (2003) and Little (2013). Whereas a full analysis of mediation requires at least three waves of data, two-wave studies offer some indication of the presence and direction of a potential indirect relationship (Taris and Kompier, 2006; Ployhart and Vandenberg, 2010; Little, 2013). Compared to cross-sectional analyses of mediation, a model based on two measurement points provides a significant improvement in inferential power due to controlling for prior levels of variables and through being able to examine the significance of the influences on the change variance of the mediator and the outcome (Cole and Maxwell, 2003; Little, 2013).

Data cleansing and descriptive data analyses were performed with IBM SPSS Statistics 22.0 (IBM Corp. Released, 2013). SEM analyses were conducted with Mplus version 7.11 (Muthén and Muthén, 1998–2013). Due to the categorical nature of the observed indicators, Weighted Least Squares Means and Variance adjusted (WLSMV) estimators were employed to determine model fit and magnitude of the relationships. Being a robust estimator, the WLSMV does not require variables to be normally distributed variables and should therefore be considered as an adequate approach for modeling categorical or ordered data (Finney and Distefano, 2006). To determine model fit, Chi-squared (χ^2^) test, root mean square error of approximation (RMSEA), Tucker-Lewis Index (TLI), and comparative fit index (CFI) were assessed. Values of RMSEA below 0.05 and values of CFI and TLI above 0.95 were considered indicative of a well-fitting model (Hu and Bentler, 1999).

Subjects were recruited at the organizational level. Thus, responses from different individuals may be correlated within organizations. Standard regression modeling is based on the assumption of independent observations and applying such statistical tests for clustered data could generate inaccurate estimates of standard errors. In the current study, we adjusted for clustered sampling by applying a sandwich estimator, obtained by the TYPE = COMPLEX option of Mplus with organization as the cluster variable (Muthen and Satorra, 1995).

## Results

### Confirmatory Factor Analyses, Descriptive Findings, and Intercorrelations

To decide whether the latent indicators of leadership, role ambiguity, role conflict, and psychological distress were empirically different, we followed a confirmatory approach that compared all possible combinations of one, two, three, and four factor measurement models at T1. Comparisons of model fit showed that the model with four correlated factors was superior to models with one, two and, three correlated factors thus indicating that fair leadership, role ambiguity, role conflict, and psychological distress represents unique constructs. The model for this four factor model exhibited good fit at both T1 [χ^2^(146, *N* = 6790) = 1120.770, *p* < 0.001, CFI = 0.984, TLI = 0.981; RMSEA = 0.031; 90% CI RMSEA = 0.030 - 0.033] and T2 [χ^2^(146, *N* = 6790) = 1034.065, *p* < 0.001, CFI = 0.982, TLI = 0.979; RMSEA = 0.030; 90% CI RMSEA = 0.028 - 0.032]. The model fit for this four-dimension model did not deteriorate when constricting factor loadings of the items across time. This provides evidence for metric invariance across time-points.

In order to assess the degree to which the reliance on a single measurement method could have affected the results (i.e., common method bias) the T1 measurement model was respecified with the addition of a common latent factor, an approach described by Podsakoff et al. (2003). Thus, an unmeasured latent factor was defined, loading on all measured items, with loadings constrained to equality to reflect a general factor that influenced all items equally, such as, e.g., personality or occasion-specific context factors at T1 that may have influenced mood or motivation. The common latent factor was not allowed to covary with the substantive factors. The results suggested little influence of such a factor. A common heuristic for evaluating the influence of common method bias is that a squared unstandardized factor loading of > 0.5 represents a problem (Eichhorn, 2014). In this case the squared loading was (-0.119)2 = 0.014.

Correlation analyses and descriptive findings are presented in **Table 1**. The descriptive findings indicated low to moderate variance in the reports of role ambiguity, role conflict, fair leadership, and distress. In line with previous research, analyses of cross-sectional data showed that fair leadership was negatively associated with role ambiguity (*r*_T1_ = -0.38, *p* < 0.001; *r*_T2_ = -0.43, *p* < 0.001), role conflict (*r*_T1_ = -0.48, *p* < 0.001; *r*_T2_ = -0.49, *p* < 0.001), and psychological distress (*r*_T1_ = -0.35, *p* < 0.001; *r*_T2_ = -0.39, *p* < 0.001) at both time points. Both role ambiguity (*r*_T1_ = 0.27, *p* < 0.001; *r*_T2_ = 0.30, *p* < 0.001) and role conflict (*r*_T1_ = 0.32, *p* < 0.001; *r*_T2_ = 0.33, *p* < 0.001) were positively associated with psychological distress in cross-sectional data.

**Table 1:** Means, standard deviations (SD), and intercorrelations for all study variables (Cronbach’s alpha in bold).

	Variable	Scale	Mean	*SD*	1	2	3	4	5	6	7	8
1	Role ambiguity T1	1–5	1.79	0.73	**0.81**							
2	Role conflict T1	1–5	2.57	0.79	0.38	**0.71**						
3	Fair leadership T1	1–5	3.97	0.83	-0.38	-0.48	**0.80**					
4	Distress T1	1–4	1.37	0.41	0.27	0.32	-0.35	**0.86**				
5	Role ambiguity T2	1–5	1.78	0.72	0.70	0.35	-0.31	0.24	**0.82**			
6	Role conflict T2	1–5	2.53	0.78	0.29	0.80	-0.34	0.24	0.45	**0.70**		
7	Fair leadership T2	1–5	3.93	0.87	-0.25	-0.35	0.58	-0.26	-0.43	-0.49	**0.83**	
8	Distress T2	1–4	1.37	0.42	0.22	0.27	-0.28	0.75	0.30	0.33	-0.39	**0.87**

*All correlations significant at the *p* < 0.001 level*.

Over time (from T1 to T2), the stabilities for the role ambiguity (*r*_T1-T2_ = 0.70; *p* < 0.001), role conflict (*r*_T1-T2_ = 0.80; *p* < 0.001), fair leadership (*r*_T1-T2_ = 0.58; *p* < 0.001), and distress (*r*_T1-T2_ = 0.75; *p* < 0.001) were moderate to high. Fair leadership at T1 was associated with decreased role ambiguity (*r*_T1-T2_ = -0.31; *p* < 0.001), role conflict (*r*_T1-T2_ = -0.34; *p* < 0.001), and distress (*r*_T1-T2_ = -0.28; *p* < 0.001) at T2. Role ambiguity at T1 was related to an increase in distress (*r*_T1-T2_ = 0.22; *p* < 0.001) and a decrease in fair leadership (*r*_T1-T2_ = -0.25; *p* < 0.001) at T2. Role conflict at T1 was associated with increased distress (*r*_T1-T2_ = 0.27; *p* < 0.001) and a decrease in fair leadership (*r*_T1-T2_ = -0.35; *p* < 0.001) 2 years later. Baseline distress was related to increased role ambiguity (*r*_T1-T2_ = 0.24; *p* < 0.001) and role conflict (*r*_T1-T2_ = 0.24; *p* < 0.001), and a decrease in fair leadership (*r*_T1-T2_ = -0.26; *p* < 0.001) at T2.

### Cross-Lagged Relationships between Fair Leadership and Role Stressors

Model comparisons of different time-lagged relationship between role ambiguity, role conflict, and fair leadership were carried out in order to test the empirical evidence for hypotheses 1a–b (fair leadership as predictor for role stressors) and 2a–b (role stressors as predictors of fair leadership). In the analyses, forward-, reverse-, and reciprocal effects models were tested and compared using a stability model as a reference. Models were compared with chi-square difference tests (the DIFFTEST option of Mplus). Model fit and comparisons for the different models are included in **Table 2**. The stability model (M1) showed acceptable fit to the data [χ^2^(111, *N* = 6790) = 940.809, *p* < 0.001, CFI = 0.984, TLI = 0.982; RMSEA = 0.022; 90% CI RMSEA = 0.020 - 0.049]. Temporal stability of the study variables was moderate to high over the 2-year period: role ambiguity (*b* = 0.69; *p* < 0.001), role conflict (*b* = 0.70; *p* < 0.001), and fair leadership (*b* = 0.60; *p* < 0.001).

**Table 2:** Results of cross-lagged structural regression between fair leadership and role stressors.

		*X*^2^	DF	CFI	TLI	RMSEA (90% C.I.)	Comparison	Δ*df*	Δ*X*^2^
M1	Stability model	940.809	111	0.984	0.982	0.022 (0.020 - 0.023)			
M2	Fair leadership T1 → Role stressors T2	930.889	220	0.985	0.982	0.022 (0.020 - 0.023)	2 vs. 1	2	37.861^∗∗∗^
M3	Role stressors T1 → Fair leadership T2	937.256	220	0.985	0.982	0.022 (0.021 - 0.023)	3 vs. 1	2	18.860^∗∗∗^
M4	Reciprocal model	920.312	218	0.985	0.982	0.022 (0.020 - 0.023)	4 vs. 1	4	59.280^∗∗∗^
							4 vs. 2	2	26.670^∗∗∗^
							4 vs. 3	2	48.965^∗∗∗^

^∗^*p < 0.05; ^∗∗^*p* < 0.01; ^∗∗∗^*p* < 0.001*.

The competing models M2, M3, and M4 were tested against the M1 stability model and against each other. As displayed in **Table 2**, the M4 reciprocal model showed significantly better fit compared to the M1 stability-, M2 forward-, and M3 reverse models. This suggests that the M4 reciprocal model gave the most valid representation of the data. In support of H1a, the structural paths in the reciprocal model (**Table 3**) showed that fair leadership at T1 was associated with decreased role ambiguity at T2 (*b* = -0.07; *p* < 0.001). Going against hypothesis H1b, no relationship was found between fair leadership and subsequent changes in role conflict (*b* = 0.02; *p* > 0.05). Supporting H2b, role conflict at T1 was negatively related to fair leadership at T2 (*b* = -0.06; *p* < 0.001). No support was found for H2a, thus indicating that role ambiguity was not related to changes in fair leadership over time.

**Table 3:** Tested associations between fair leadership and role stressors in the M4 Reciprocal model.

Relationship	Standardized estimate (SE)
Fair leadership T1 – fair leadership T2	0.535^∗∗∗^ (0.013)
Fair leadership T1 – role ambiguity T2	-0.074^∗∗∗^ (0.009)
Fair leadership T1 – role conflict T2	0.020 (0.019)
Role ambiguity T1 – role ambiguity T2	0.645^∗∗∗^ (0.010)
Role ambiguity T1 – fair leadership T2	-0.026 (0.014)
Role conflict T1 – role conflict T2	0.719^∗∗∗^ (0.027)
Role conflict T1 – fair leadership T2	-0.063^∗∗∗^ (0.014)

^∗^*p < 0.05; ^∗∗^*p* < 0.01; ^∗∗∗^*p* < 0.001*.

### Psychological Distress as a Mediator

To determine the impact of psychological distress as a mediator of the relationships between fair leadership and subsequent changes in role stressors, as well as the impact of role stressors on subsequent changes in fair leadership, we followed the recommendations for testing a half-longitudinal mediation model by Cole and Maxwell (2003) and Little (2013).

As for psychological distress as a mediator between fair leadership and the role stressors (H3a and H3b), the findings did not support any indirect effects. Controlling for prior levels of psychological distress, gender, age, and skill level, fair leadership (path a: *b* = -0.01; *p* > 0.05) at T1 was not related to changes in distress from T1 to T2. Distress at T1 was related to changes in role ambiguity (path b: *b* = 0.05; *p* < 0.001) over time, but not role conflict (path b: *b* = -0.02; *p* > 0.05) at T2. As the non-significant association between fair leadership and distress violates the assumption of causal relationship between predictor and mediator variable, the analyses show that psychological distress does not function as a mediator of the relationship between fair leadership as predictor variable and role ambiguity and role conflict as an outcome variable.

In a similar manner, we found no evidence for distress as a mediator of the reverse associations between the role stressors and subsequent fair leadership. The analyses of the associations proposed in H4a and H4b showed that neither role ambiguity (path a: *b* = -0.006; *p* > 0.05) nor role conflict (path a: *b* = 0.03; *p* > 0.05) at T1 were related to changes in distress from T1 to T2, again violating the assumptions about a relationship between predictor and mediator variable. Distress at T1 was significantly related to a reduction in fair leadership from T1 to T2 (path b: *b* = -0.081; *p* < 0.001).

## Discussion

The current study extends previous research by examining time-lagged associations between fair leadership and exposure to role stressors and by proposing psychological distress as a potential intervening variable that would mediate both forward and reverse associations between the main study variables. After adjusting for stability in the outcome variable, age, gender, skill level, and clustered sampling, the findings showed that leadership fairness was associated with a significant decrease in role ambiguity, but not with role conflict, over the 2-year study period. As for the impact of role stressors on subsequent reports of leadership, role conflict, but not role ambiguity, was significantly related to a decrease in fair leadership over time. Against our expectations, psychological distress did not mediate the association between fair leadership and subsequent role ambiguity and role conflict. Similarly, no mediating effect of psychological distress was found in the association between the role stressors and later reports of fair leadership.

While there are no previous time-lagged studies that have examined associations between leadership fairness and role stressors, the significant negative relationship between fair leadership and role ambiguity is in line with previous longitudinal research on related indicators such as transformational leadership (Schaubroeck et al., 1993; Moyle, 1998; Nielsen et al., 2008). This finding suggest that fair leaders may reduce the uncertainty about the stressors, behavior, and consequences that are associated with a particular role among subordinates. On the other hand, as fair leadership was not related to changes in role conflict over time, leadership fairness seems to have little impact on whether an employee experiences incongruence between different roles. Hence, leadership fairness may therefore be more relevant with regard to some aspects of the job situation than to others. An explanation for the differential impact of fair leadership on role ambiguity and role conflict may be that clarification of roles may be more dependent upon a leader who distribute work tasks and resources in a fair manner, whereas the occurrence of role conflict may be more dependent upon organizational factors or upon the nature of work being performed.

Extending previous findings on fair leadership and work factors, and also adding to the scarce literature on prospective research on leadership in general, our findings showed that the experience of role conflict at the workplace was associated with perceiving the immediate leader as less fair over time. This results show that leadership should not solely be considered as a causal factor that influence the job situation of subordinates, but that exposure in the job situation may also influence how workers perceive their immediate leader. In doing so, the findings of this study support the arguments for a follow-centered perspective on leadership as discussed by Shamir and colleagues (e.g., Howell and Shamir, 2005; Shamir, 2007).

Based on the rosy/gloomy perception mechanism, we proposed psychological distress as a potential intervening variable that could explain the reciprocal relationships between leadership and the investigated role stressors. However, the results did not support any indirect associations through distress. There may be several explanation for these non-significant findings. First, it may simply be that distress does not mediate the relationships and that a rosy/gloomy perception is not valid a mechanism for explaining how the variables are interrelated. If this is the case, other potential mediators, such as for instance positive and negative affect, should be examined in upcoming research. However, issues related to the research design may also have caused the non-significant associations. In the current study, we employed a 2-year time-lag between the measurement points. While a 2–3 year lag has been found to provide the strongest associations between stressors and strain in prospective research (Ford et al., 2014), it may still be that other results could have been obtained with shorter or longer time frames. On the one hand, fair leadership may have short-term effects that attenuate over a 2 year period. On the other hand, as a mediation process may need time to develop, there could also be sleeper effects that first emerge over a longer time period (Zapf et al., 1996).

We used a half-longitudinal design to examine mediation in this study. This design provides a significant improvement in inferential power compared to a cross-sectional test of mediation by enabling control for prior levels of the variables and examination of the significance of the influences on the change variance of the mediator and the outcome (Little, 2013). However, this design has some untestable assumptions (e.g., stationarity) that limits its contribution (Taris and Kompier, 2006; Little, 2013). A true longitudinal design with three or more measurement points will provide a more thorough test of indirect associations and could therefore yield different results.

Finally, the associations between fair leadership, distress, and role stressors could also be explained by unmeasured third variables. For instance, a strong fairness climate or high quality leader-member exchange may reduce role stressors and distress, while simultaneously increasing perceptions of fair leadership. Alternatively, the variables may be influenced by personality traits like conscientiousness or neuroticism. A suggestion for future research is to include stronger tests of the proposed associations by including such third variables. Nonetheless, omission of significant third variables will always be a limitation of survey research as it is impossible to account for all potential confounders. Manipulating third variables in a field experiment may therefore be more functional with regard to establishing causality (Schaubroeck et al., 1993). For instance, after a baseline assessment, one could train leaders to be fairer, and then subsequently reassess employees’ perceptions of role stressors. Similarly, one could create an intervention to lessen role stressors and then assess whether and how this affects fair treatment by leaders.

Although psychological distress did not mediate the associations between fair leadership and role stressors, we found that distress was associated with an increase in role ambiguity and a decrease in leadership fairness over time. Hence, distress may still be an important precursor to both working conditions and justice. The association between distress and subsequent reports of fair leadership is in line with previous research on justice (Birkeland et al., 2016). For instance, in their three sample time-lagged study of relationships between justice perceptions (i.e., distributive justice, interactional justice, interpersonal justice, informational justice, and procedural justice) and depression using 3–6 month time-lags, Lang et al. (2011) found consistent significant evidence for depression as a precursor to justice perceptions despite variations in context and justice dimensions across samples. Similar to the findings of our study, the opposite effects of organizational justice perceptions on depressive symptoms were not significant for any of the justice dimensions (Lang et al., 2011).

### Methodological Strengths and Limitations

The present study examined bidirectional relationships between the role stressors, and fair leadership in a large longitudinal sample. The sample comprised employees from a variety of different enterprises and was adjusted for clustered sampling. Psychometrically sound measurement instruments were used to measure the study variables. The response rate was above the average level established for organizational surveys (Baruch and Holtom, 2008). Attrition analyses indicated that the cohort was more or less representative for the overall baseline sample on the study variables. The findings should therefore be generalizable to the larger population. Despite the large sample, participating organizations were recruited through convenience sampling methods, something that limits the external validity of the findings. However, all employees in the participating organizations were invited to participate in the survey. The sample can therefore be considered as a probability sample at the individual level (Ilies et al., 2003).

Because the questionnaire instruments were self-report measures, the study could be influenced by bias such as response-set tendencies and social desirability. However, it has been argued that the QPS_Nordic_ instrument used to assess role stressors and fair leadership are fairly insensitive to respondents’ emotions or personality dispositions in that subjects report frequency of occurrence rather than degrees of agreement or satisfaction and items do not address issues that are inherently negative or positive (Christensen and Knardahl, 2012).

The use of self-report measures implies a risk of common method variance (CMV), i.e., “variance that is attributable to the measurement method rather than to the constructs the measures represent” (Podsakoff et al., 2003, p. 879). In the current study, several ex ante strategies were used to reduce the risk of CMV (Chang et al., 2010). First, respondents were assured of the anonymity and confidentiality of the study, that there were no right or wrong answers, and that they should answer as honestly as possible. In addition, great care was taken to systematically examine the construction of items in order to ensure that ambiguous, vague and unfamiliar terms were not included, and that the questionnaire as a whole and the individual items were formulated as concisely as possible. The indicator of psychological distress had different scale end points than the indicators of leadership and role stressors. This should reduce method biases caused by commonalities in scale endpoints and anchor effects. Finally, the use of a time-lag between the measurement of the independent and dependent variables in the current study may have contributed to reduce the risk of CMV. Supporting an effect of the ex-ante strategies, the ex-post tests of CMV used in this study provided no evidence of this kind of bias.

It should be noted that the scales of role conflict, role ambiguity, and fair leadership were comprised of three items each. Although the utilized scales are thoroughly tested and validated (Dallner et al., 2000; Wannstrom et al., 2009), they measure relatively complex constructs. Hence, it may be questioned whether three item indicators can capture all the variation in fair leadership and role conflict and ambiguity. Future research should therefore replicate our findings using instruments that are more specific and sensitive.

The established cross-lagged effect sizes were relatively small (-0.06 to -0.07). While these estimates may seem limited, a small effect size should be expected both from a content and a methodological point of view (Zapf et al., 1996). There are many factors that could influence both role stressors and fair leadership and when adjusting for the stability in the outcome variable it is highly unlikely that one single predictor variable alone should explain the lion’s share of the variance in the outcome. Actually, a recent meta-analysis have shown that effect sizes in the range of ±0.05 – 0.10 is the average in full-panel time-lagged studies (Ford et al., 2014). In addition, even though an effect size is small, the impact on individuals and organizations may still be substantial (Cortina and Landis, 2009).

## Implications and Conclusion

Despite a wealth of research on leadership, there is a shortage of studies employing temporal research design. Hence, this study is among the first to show that leadership can influence the working conditions of subordinates over time. Specifically, we found that subordinates who perceive their immediate leader as fair reported a decrease in role ambiguity, but not role conflict over a 2-year time-period. Hence, the findings add support to the hypothesis that leadership is an antecedent to levels of role ambiguity in an organization (Nielsen et al., 2008; Lawrence and Kacmar, 2012). In addition to confirming leadership as a potential causal factor, a novel finding from this study is that subordinates who experienced high levels of role conflict perceived their immediate leader as less fair over the study period. A main implication of the current study is therefore that theoretical models of organizational behavior, as well as research on leadership, also must take into consideration a reverse relationship where exposures at the workplace also influence how subordinates perceive their leader. A practical implication of our findings is the importance of addressing role stressors in organizations since exposed workers are more likely to experiencing unfairness in the leader-member exchange. Organizations should therefore prioritize setting clear goals, provide employees with necessary information for conducting a given work task, and thereby help workers understand their roles at the workplace.

We hypothesized that psychological distress was a mediating mechanism that would explain how fair leadership and role stressors were interrelated. As our findings did not support this assumption, there is a need for research that can replicate our study in other samples and settings, and with modified research designs, as well for time-lagged studies examining alternative mediators that can explain how leadership may influence role stressors as well as how role stressors can influence leadership. Whereas a time-lagged design does not confirm any form of causality, the design does satisfy one essential condition for a cause and effect associations in that the predictor variables are measured prior to the outcome variable (Shamir, 2011). Consequently, an advantage of the full-panel prospective design is that it points to whether fair leadership actually is related to changes in role stressors. Furthermore, the full panel design makes it possible to determine the existence of any reverse associations that add to the understanding of bidirectional relationships between study variables.

## Author Contributions

MN in initiated the study, conducted analyses, and was responsible for writing the manuscript. JC participated in the idea development, conducted analyses, wrote results, and read all versions of the manuscript. LF participated in the idea development, contributed to the structure and content, and read all versions of the manuscript. SK was responsible for the data collection, participated in the idea development, contributed to the structure and content, and read all versions of the manuscript.

## Conflict of Interest Statement

The authors declare that the research was conducted in the absence of any commercial or financial relationships that could be construed as a potential conflict of interest.
